# Depicting the RNA Virome of Hematophagous Arthropods from Belgrade, Serbia

**DOI:** 10.3390/v12090975

**Published:** 2020-09-02

**Authors:** Maja Stanojević, Kun Li, Gorana Stamenković, Bojan Ilić, Milan Paunović, Branislav Pešić, Ivana Đurić Maslovara, Marina Šiljić, Valentina Ćirković, Yongzhen Zhang

**Affiliations:** 1 Faculty of Medicine, Institute of Microbiology and Immunology, University of Belgrade, 11000 Belgrade, Serbia; maja.stanojevic@med.bg.ac.rs (M.S.); marinasiljic@gmail.com (M.Š.); valentinanikolic85@gmail.com (V.C.); 2 Department of Zoonoses, National Institute for Communicable Disease Control and Prevention, Changping, Beijing 102206, China; likun@icdc.cn; 3 Department for Genetic Research, Institute for Biological Research “Siniša Stanković”—National Institute of Republic of Serbia, University of Belgrade, 11000 Belgrade, Serbia; gorana.stamenkovic@ibiss.bg.ac.rs; 4 Department of Animal Development, Faculty of Biology, Institute of Zoology, University of Belgrade, 11000 Belgrade, Serbia; bojan.ilic@bio.bg.ac.rs; 5 Natural History Museum in Belgrade, 11000 Belgrade, Serbia; milan.paunovic@nhmbeo.rs; 6 Institute for Biocides and Medical Ecology, 11000 Belgrade, Serbia; banekomarci@gmail.com (B.P.); ivanaveterinar@gmail.com (I.Đ.M.); 7 Shanghai Public Health Clinical Center & Institute of Biomedical Sciences, Fudan University, Shanghai 200432, China

**Keywords:** hematophagous arthropods, RNA virome, pathogens

## Abstract

Hematophagous arthropods are important vectors for zoonotic pathogens. To date, a huge number of viruses have been identified in these arthropods, with a considerable proportion of them being human pathogens. However, the viromes of hematophagous arthropods are still largely unresearched. In this study, a number of arthropods were collected from Belgrade, Serbia including mosquitoes, ticks and bedbugs. The viromes of these arthropods were identified and characterized using Illumina MiSeq sequencing. In total, 21 viruses belonging to 11 families were characterized, with 11 of them representing novel species. These results may contribute to our knowledge of RNA viruses in arthropods and the discovery of novel human pathogens.

## 1. Introduction

Phylum Arthropoda is estimated to contain as many as 5–10 million species, which accounts for approximately 80% of the Kingdom Animalia [1]. They play an important role in marine, freshwater, land and air ecosystems and because of their extensive diversity they are a natural reservoir for a diverse range of viruses. Until now, over 1500 arthropod virus species have been reported, revealing a substantial genomic heterogeneity that is still largely underestimated [2,3,4].

As currently estimated, more than two-thirds of the viral pathogens infecting humans are of zoonotic origin [5]. Hematophagous arthropods (e.g., ticks, mosquitoes, midges, sandflies, fleas, etc.) are important vectors for a multitude of zoonotic pathogens that are closely associated with human disease. Viruses transmitted to humans via arthropod vectors (e.g., dengue virus, West Nile virus, yellow fever virus, Japanese encephalitis virus, etc.) are known as arthropod-borne viruses (arboviruses) [6]. More than 100 human pathogenic arboviruses have been characterized and are classified into seven main families (*Flaviviridae*, *Togaviridae*, *Nairoviridae*, *Phenuiviridae*, *Reoviridae*, *Rhabdoviridae*, and *Orthomyxoviridae*) [6,7,8]. Over the past few decades, these viruses have posed a considerable threat to public health worldwide, and have caused millions of infections and deaths [9,10]. Although great efforts have been made, there is no doubt that a large number of uncharacterized viruses with the potential to become human pathogens still exist.

The meta-transcriptomics approach has enabled the rapid discovery of novel viruses, including those potentially infectious to humans. The viromes of some hematophagous arthropods have been well characterized using metagenomic methods [11,12,13,14,15,16], and lots of novel arthropod-specific viruses and vertebrate viruses have been identified. However, the viromes of various hematophagous arthropods are still poorly understood, especially those from Southern Europe. In order to understand the genomic biodiversity of viruses and identify potential arthropod-associated human pathogens, we comprehensively characterized and analyzed the viromes of multiple species of hematophagous arthropods sampled from Serbia, mostly in the capital city of Belgrade.

## 2. Methods

### 2.1. Collection and Processing of Arthropods

During June to September 2016, arthropods were collected from 27 sites in Serbia, most of which were within the city of Belgrade and its near surroundings (20.40° E, 44.59° N). The arthropods were immediately stored at −80 °C and then transported to China CDC at room temperature in DNA/RNA Shield (Zymo Research, Irvine, CA, USA) until RNA/DNA extraction. All arthropod samples were morphologically identified by an experienced technician, and this was subsequently confirmed by amplifying the mitochondrial 18S ribosomal RNA (18S rRNA) gene sequences and comparing them to the reference sequences from GenBank.

### 2.2. Sample Processing and RNA Extraction

The arthropod samples were removed from the DNA/RNA shield and then washed three times with phosphate-buffered saline (PBS). The collected arthropods were grouped into 39 batches based on species, collection place and date (360 mosquitoes were grouped in 18 batches, 37 ticks in 8 batches and 118 bedbugs/other arthropods in 13 batches) (Supplementary Materials, Table S1). The samples were homogenized into suspension in an 0.8 mL PBS solution on ice and then subjected to 2500× *g* centrifugation for 5 min. The supernatant was collected for RNA extraction. The QIAamp viral RNA Mini Kit (Qiagen GmbH, Hilden, Germany) was used for RNA extraction according to the manufacturer’s instructions.

### 2.3. RNA-Seq Sequencing, Reads Assembly, Virus Sequences Discovery and Confirmation

The total RNA of mosquitoes, ticks and bedbugs/other arthropods was subjected to RNA-seq library preparation following the standard Illumina protocol. The RNA was fragmented after the removal of rRNA using the Agilent 2100 Bioanalyzer, reverse-transcribed, placed in an adaptor, purified and examined by the ABI StepOnePlus Real-Time PCR System. Based on the hosts, the extracts were further merged into 3 pools for RNA-seq library construction and sequencing. The resulting reads were quality trimmed and then assembled into 263,883–355,228 contigs using the Trinity program. The genome of the hosts was not eliminated. The contigs were translated and compared to reference protein sequences of all known RNA viruses to search for virus sequences using Blastx. Sequences with e-values less than 1 e^−5^ were retained. The resulting sequences were then merged by identifying unassembled overlaps between neighboring contigs or within a scaffold using SeqMan implemented in the Lasergene software package v7.1 (DNAstar, Madison, WI, USA). Also, procedures for screening negative controls and elimination of the genome of arthropods hosts were not included in process. Finally, based on the metagenomic results, primers were designed for the nested RT-PCR protocol for all potential viral sequences obtained and used to screen the initial sample batches in order to examine which unit contained the target sequences (Supplementary Materials, Table S2).

### 2.4. Acquisition of the Coding-Complete Sequences or RdRp Genes

Some of the sequences obtained from the metagenomic approach were shorter than expected. To obtain complete CDS sequences or RNA dependent RNA polymerase (RdRp) genes, consensus primers were designed by comparing the obtained sequences with known viral sequences (Supplementary Materials, Table S3). After nested RT-PCR or semi-nested RT-PCR, the products were analyzed on 1.0% agarose gels. Single fragments were recovered and purified using an Minibest Agarose Gel DNA Extraction Kit (TaKaRa Bio, Kusatsu City, Japan). The purified products were then ligated into pMD19-T Vector Cloning Kit (TaKaRa Bio, Japan) and transformed into *E. coli* DH5α cells. Clones were randomly picked and sent for Sanger DNA sequencing (Sangon Biotechnology Company, Shanghai, China).

### 2.5. Phylogenetic Analysis

Viral RdRp sequences obtained in this study were aligned with the corresponding homologues of reference viruses (Supplementary Materials, Table S4). The sequence alignment was limited to conserved domains, with ambiguously aligned regions removed using Gblocks (v0.91b) [17]. Phylogenetic trees were inferred using the maximum likelihood method (ML) implemented in PhyML version 3.0 [18]. To estimate the support value for individual nodes, 1000 bootstrap replicates were obtained under the same procedure. All phylogenetic trees were mid-point rooted.

## 3. Results

### 3.1. Discovery of Divergent RNA Viruses in Arthropods

From June to September 2016, a total of 515 arthropods were sampled from 27 sites in Serbia (Table 1). They represent nine species and most of them are hematophagous arthropods (471/515) including ticks, mosquitoes, bedbugs, fleas, etc. RNA viromes in these arthropod samples were sequenced using Illumina MiSeq. About 106,073,982 reads were generated from three RNA sequencing libraries, which contained 37 *I. ricinus* ticks, 360 mosquitoes and 118 bedbugs/fleas/other arthropods, respectively. The reads were de novo assembled and then Blastx comparisons against sequences of RNA viruses were performed. We identified putative viral sequences and determined complete coding genome sequences or RdRp sequences where possible. In total, this study identified 21 viral species in the studied arthropods, based on CDS or near complete amino acid sequences of the RdRp. These data provide evidence for at least 10 known species and 11 potentially new species of RNA viruses, defined as those whose RdRp sequences share less than 25% amino acid identity with existing species (Table 2, Table S5). Although we did not perform PCR on DNA extract to exclude the possibility of these viruses being endogenous viral elements (EVEs), these viruses are not likely to be EVEs as they are present as complete genomes or CDSs without any interruptions. These viruses represent a variety of RNA virus taxa with +ssRNA, −ssRNA, and dsRNA genome and are specific to invertebrates, vertebrates or even to fungi and plants. Taxonomically, these viruses belong to the families/orders of *Bunyavirales*, *Orthomyxoviridae*, *Mononegavirales*, *Picornavirales*, *Reoviridae*, *Luteoviridae*, *Narnviridae*, *Chrysoviridae* and *Virgaviridae.* Finally, all of these virus sequences were successfully confirmed by RT-PCR.

### 3.2. Diverse Viruses Identified in Mosquitoes

A variety of viral sequences were identified in the datasets generated from mosquitoes, including those that fell within the families and orders of *Mononegavirales*, *Bunyavirales*, *Narnaviridae*, *Picornavirales*, *Virgaviridae*, *Reoviridae* and *Luteoviridae*. In total, nine tentative novel viral species were identified in mosquitoes, suggesting that some of the viruses were highly prevalent in mosquitoes. In particular, Serbia narna-like virus 1 str. 85061, Serbia virga-like virus 1 str. 88617 and Serbia picorna-like virus 1 str. 71282 appeared in all except one of the 18 mosquito batches. These results highlight the very common occurrence of some viral infections in the studied population of *Culex pipiens*.

**Figure 1:**
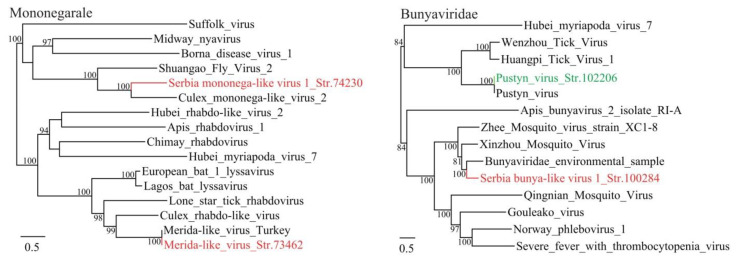
Phylogenetic analysis of -ssRNA viruses including Mononegarales and Bunyavirales. Phylogenetic trees were inferred using the ML method, with 1000 bootstrap replicates and mid-point rooting. Red: viruses from mosquitoes (*Culex Pipiens*), green: virus from ticks (*Ixodes ricinus*).

*Mononegavirales* is a large, diverse order of viruses that have ss −RNA genomes. In this study, two members of the *Mononegavirales* order were identified in mosquitoes, one of which (Merida-like virus str. 73462) shows 99% aa identity with the Merida-like virus, from the *Rhabdoviridae* family. The Merida-like virus was initially identified in *C. pipiens* mosquitoes from Thrace/Anatolia, Turkey, which is located over 1000 km from Belgrade [19]. This finding may suggest the common occurrence and high conservation of this virus in *Culex* mosquitoes. Another mononega-like virus was tentatively assigned as a new virus species and nominated as the Serbia mononega-like virus 1. The proposed Serbia mononega-like virus 1 is predicted to have a 12,933 nt long genome, presenting a single ORF between coordinates 761–10,147 and encoding a putative 3128 aa long polyprotein. The Serbia mononega-like virus 1 is genetically related to the Culex mononega-like virus 2 (CuMV2) identified in western Australia (Identity: 56.6% at the RNA level, and 41% at the predicted protein level), which is currently unclassified but assigned to the order of *Mononegavirales* [20]. In the phylogenetic tree, the Serbia mononega-like virus 1 and CuMV2 cluster together and form a distinct clade, indicating that they may represent a group of widely distributed *Culex*-borne mononega-like viruses (Figure 1).

*Virgaviridae* is a family of ss +RNA viruses known to infect plants [21]. In this study, two virga-like viruses were identified in mosquitoes. One shows 91% amino acid identity to Hubei virga-like virus 2 identified in mosquitoes sampled from China [3], while the other shows as low as 31% amino acid identity to Loreto virus with a 43% coverage [22], suggesting that it represents a novel species (Serbia virga-like virus 1). Genome structure analysis indicates that both length and location of ORFs are similar to those of other Virgaviruses. Although the detection rate is quite high (17/18, 94.44%), it is possible that these sequences are not from the mosquito themselves but from their food such as plant nectar or fruit juices.

**Figure 2:**
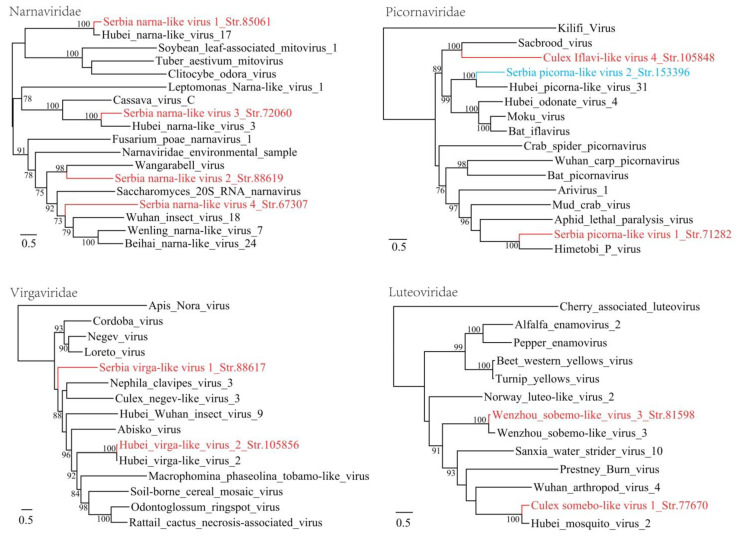
Phylogenetic analysis of +ssRNA viruses including Narnaviridae, Picornaviridae, Virgaviridae and Luteoviridae. The phylogenetic trees were inferred using the maximum likelihood (ML) method, with 1000 bootstrap replicates and mid-point rooting. Red: viruses from mosquitoes (*Culex Pipiens*), blue: virus from bedbugs (*Cimex lectularius*).

*Narnaviridae* is a family of ss +RNA viruses with a genome normally between 2.3–3.6 kb [23]. In this study, four novel members of the *Narnaviridae* were identified in mosquitoes and the complete or nearly complete genome sequences were obtained. Of these, the Serbia narna-like virus 1 str. 85061 shows extremely high prevalence with a 94.74% positive rate in all batches, while the other three virus species have a positive rate of 5.26% each. Fungi are believed to be natural host for narnaviruses [23]. In recent years, a number of narnaviruses has been identified in arthropods, nevertheless, as they are often co-detected with a group of fungal pathogens, it may be considered more plausible for them to be truly associated with fungi than with mosquitoes themselves [24]. In the phylogenetic tree, Serbia narna-like virus 1 str. 85061 and Serbia narna-like virus 3 str. 72060 closely clustered with Hubei narna-like virus 3 and Hubei narna-like virus 17 in mosquitoes sampled from China [3], respectively (Figure 2). Meanwhile, the Serbia narna-like virus 2 str. 88619 strain forms a distinct lineage in the tree with a highest 30% amino acid identity and 90% coverage to the Wangarabell virus identified in ticks from Australia [13].

The complete genome sequences of two picornaviruses were obtained. One is a Culex Iflavi-like virus 4 which was firstly identified in California, the USA [14]. The amino acid identity between these two picornaviruses is as high as 98% and the positive rate in Serbia mosquito batches is 36.84%, suggesting the widespread distribution of this virus in a wide range of geographic areas. Another picornavirus named as Serbia picorna-like virus 1 str. 71282 in this study is mostly related to Himetobi P virus that was found to infect *Laodelphax striatellus* planthopper in Japan [25]. The highest 41% amino acid identity and 54% coverage indicate that it represents a new picorna-like virus species.

**Figure 3:**
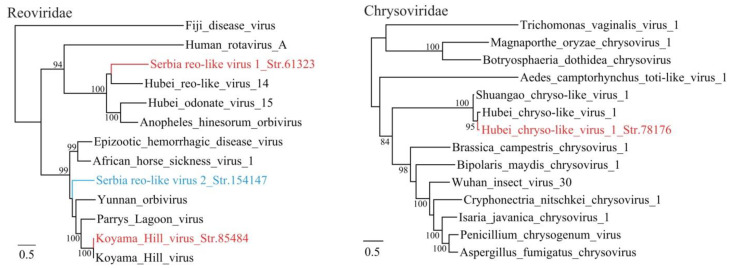
Phylogenetic analysis of dsRNA viruses including Reoviridae and Chrysoviridae. The phylogenetic trees were inferred using the ML method, with 1000 bootstrap replicates and mid-point rooting. Red: viruses from mosquitoes (*Culex Pipiens*), blue: virus from bedbugs (*Cimex lectularius*).

A novel bunya-like virus named Serbia bunya-like virus 1 was identified in the mosquito samples. The L segment has a length of 7649 nt, encoding a single ORF of putative 1995 aa long RNA-dependent RNA polymerase. It shows highest identity of 57% with bunyaviruses identified from *Culex pipiens* in northern California, USA [24]. Furthermore, one Reo-like virus (Serbia Reo-like virus str. 61323) strain was identified, which may also represent a novel virus species (Figure 3). The sequences of some *Chrysoviridae* viruses were also identified from the sequence dataset obtained from mosquitos.

### 3.3. Viruses Identified in Ticks, Bedbugs and Fleas

Virus sequences belonging to *Luteoviridae*, *Picornaviridae*, *Bunyavirales* and *Mononegavirales* were identified from the *Ixodes ricinus* ticks. The complete sequence of a Pustyn virus strain, which belongs to *Nairoviridae* was identified. It shows as high as 99% aa identity to the Pustyn virus identified from the Nizhniy Novgorod district, which is located 2000 km from Belgrade, suggesting the extremely high conservation of this virus. Besides, partial RdRp sequences of a mononega-like virus, a bunya-like virus and a Falcovirus were identified, which show 51%, 52% and 99% identities to the Norway mononegavirus 1, Hubei odonate virus 9 and Falcovirus A1 [22,26], respectively. However, the complete RdRp sequences were not obtained in this study.

**Figure 4:**
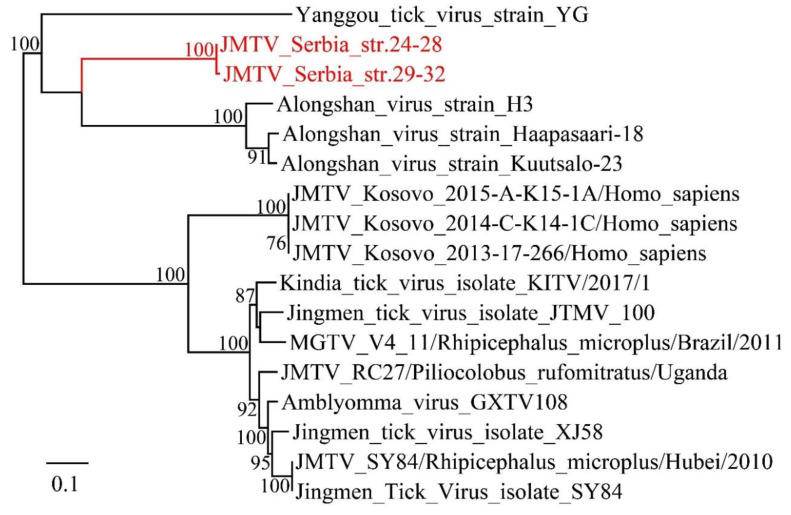
Phylogenetic analysis of the Jingmen tick viruses. The phylogenetic trees were inferred using the ML method, with 1000 bootstrap replicates and mid-point rooting.

Furthermore, it is noteworthy that a Jingmen virus was identified in three ticks pools. The Jingmen virus is a distinct group of tick-borne viruses that possess a segmented genome but show partial sequence identity to unsegmented viruses [27]. In this study, the complete ORF of RdRp was obtained. Phylogenetic analysis indicates that this Jingmen strain is mostly related to the Alongshan virus with 88% aa identity, which has been shown to infect humans and cause febrile symptoms in China [28] (Figure 4). The Jingmen virus has recently been reported in the Balkans in Crimean-Congo hemorrhagic fever patients from Kosovo [29]. However, the newly described Jingmen virus Belgrade strain has only 80% aa identity to the Kosovo strain. In sum, this result indicates the high genetic diversity of Jingmen viruses in the Balkan Peninsula, moreover, it implies that this Jingmen virus strain might have the potential to cause human diseases.

In the current study, two novel viruses were identified in *Cimex lectularius* bugs. A picorna-like virus named Serbia picorna-like virus 2 Str. 153396 was identified, which shows the highest identity to Hubei picorna-like virus 31 from Odonata with 41% aa identity [3]. A reo-like virus named Serbia reo-like virus 2 Str. 154147 shows the highest 49% aa identity to several Orbiviruses, such as the Parry’s Lagoon virus identified from *Culex annulirostris* mosquitoes and the Big Cypress virus from *Psorophora columbiae* mosquitoes [30]. In the phylogenetic tree, this virus falls into the orbivirus cluster, indicating that it is a novel species of the genus *Orbivirus*, family *Reoviridae*. Orbiviruses are mainly harbored and transmitted by haematophagus arthropods, such as ticks, mosquitoes and sandflies (Figure 3). Some of them cause animal diseases (bluetongue virus, African horse sickness virus, epizootic hemorrhagic disease virus, etc.) [31]. Notably, IgM and IgG against Yunnan orbivirus have been detected in one patient in China, suggesting that human infections by orbivirus may occasionally occur [32]. In this study, the Serbia reo-like virus 2 Str. 154147 is the first report of an Orbivirus identified from *Cimex* bed bugs. Its pathogenicity to humans and animals has yet be studied.

## 4. Discussion

In this study, we used a meta-transcriptomics approach to characterize the viromes in hematophagous arthropods including mosquitoes, ticks, bedbugs and fleas. The complete or near complete RdRp sequences of 21 RNA viruses were identified and phylogenetically analyzed, and 11 of these represent novel virus species. These virus species fell into known families and orders including *Mononegavirales*, *Bunyavirales*, *Narnaviridae*, *Picornavirale*, *Virgaviridae*, *Reoviridae*, and *Luteoviridae*. The studies herein suggest that although much work has been done on arthropod viral vectors, the realm of new viruses in them still needs to be characterize. The study provided an insight into the rich genetic and phylogenetic diversity of the hematophagous arthropod viromes. These arthropods, especially mosquitoes and ticks, are some of the most important viral vectors in the world and are closely related to human diseases. Serbia, situated in the Balkan Peninsula in Southern Europe, is known as an endemic region for arthropod borne pathogens [33]. Several arboviruses are known to be present and circulating in Serbia, e.g., the Crimean-Congo haemorrhagic fever virus (CCHFV) was reported for the first time in several decades, while the West-Nile virus (WNV) was detected less than 10 years ago in 2012, when the biggest seasonal WNV outbreak in Europe was reported in Serbia, mainly in Belgrade [34,35]. Notably, few known human or animal arboviruses were identified in this study, although previous studies detected West Nile virus in nearly 10% of studied mosquito pools in 2014 and 2015 [36]. The pathogenicity of the novel viruses identified in this work is still to be determined in further study. In addition, it is noteworthy that a Jingmen virus strain was characterized in *Ixodes* ticks, which shows the highest 88% aa identity to the Alongshan virus identified in a febrile patient in China [28] and 80% aa identity to a Jingmen virus strain from hemorrhagic fever patients from Kosovo [29]. The prevalence of the Jingmen virus in tick batches was as high as nearly 40%, which indicates the potential public health risk of this virus in Serbia.

Of the RNA viruses discovered here from mosquitoes, four were from families of (+) ssRNA viruses (*Luteoviridae*, *Virgaviridae*) currently only known to infect plants and one was from viruses specific to animals (Falcovirus). Since both male and female mosquitoes feed on plant nectar and fruit juices, and females also feed on the blood of animals, it is possible that some of these sequences are not mosquito-infecting viruses but are of plant or animal origin. Furthermore, in mosquitoes and ticks, some members of *Narnaviridae* were identified, which are naturally harbored by fungi. As was previously illustrated, it is more plausible that these sequences are from fungal pathogens infecting arthropods, instead of the arthropods themselves [24]. However, the extremely high abundance of Serbia narna-like virus 1 suggests the possibility that they are directly associated with the arthropod hosts.

Arthropods have been considered as the major reservoir hosts for many RNA viruses in vertebrates and plants. In recent years, an enormous range of viruses has been identified in arthropods, many of which appear to be ancestral to recognized virus species [3,4]. The amazing number and genetic diversity suggest that our knowledge of viromes in arthropods is still superficial or even primitive. Our work identified 11 novel viruses located in various positions of the phylogenetic tree, which further contributes to a better understanding of the biodiversity and evolutionary history of arthropod viruses. It is also interesting that some of the arthropod viruses are widely distributed geographically and have very little divergence. For example, the Hubei_virga-like_virus_2_Str. 105856 and Merida-like_virus_Str. 73462 show >99% identity to virus strains identified in China and Turkey, respectively, which are thousands of kilometers in geographical distance from Belgrade. This low divergence across large geographical distances may suggest the close association of these viruses to their arthropod hosts throughout evolutionary history.

## Figures and Tables

**Table 1:** Summary of the sampling sites, species and sequenced data.

Location	Coordinates	Species	Pool Size	No. of Sequenced Reads	Average Length of Reads
Belgrade	N44.759–N44.881 E20.244–E20.625	*Culex pipiens L*	360	43699119	150
Belgrade and surrounding areas	N42.710–N45.455 E19.220–E22.342	*Ixodes ricinus*	37	28141701	150
Belgrade and surrounding areas	N44.690–N44.935 E20.450–E21.136	*Cimex lectularius* and other arthropods	118	34233162	150

**Table 2:** Novel viruses identified in this study.

Provisional Name	Accession Number	Host	Taxon	Best Hit	Coverage (%)	Identity (%)	Sequence Length	Obtained Region
Serbia mononega-like virus 1_Str. 74230	MT822181	*Culex pipiens*	*Mononegavirale*	Culex mononega-like virus 2	43	41	12,933	Complete CDS
Serbia bunya-like virus 1_Str. 100284	MT822182	*Culex pipiens*	*Bunyavirales*	Bunyaviridae environmental sample	95	57	7649	Complete RdRp
Serbia narna-like virus 1_Str. 85061	MT822183	*Culex pipiens*	*Narnaviridae*	Hubei narna-like virus 17	83	71	3280	Complete RdRp
Serbia narna-like virus 2_Str. 88619	MT822184	*Culex pipiens*	*Narnaviridae*	Wangarabell virus	90	30	5269	Complete RdRp
Serbia narna-like virus 3_Str. 72060	MT822185	*Culex pipiens*	*Narnaviridae*	Hubei narna-like virus 3	89	48	2685	Complete RdRp
Serbia narna-like virus 4_Str. 67307	MT822186	*Culex pipiens*	*Narnaviridae*	Hubei narna-like virus 16	70	44	2889	Complete RdRp
Serbia picorna-like virus 1_Str. 71282	MT822187	*Culex pipiens*	*Picornavirale*	Himetobi P virus	56	41	9132	Complete CDS
Serbia virga-like virus 1_Str. 88617	MT822191	*Culex pipiens*	*Virgaviridae*	Loreto virus	43	31	10,501	Complete CDS
Serbia reo-like virus 1_Str. 61323	MT822189	*Culex pipiens*	*Reoviridae*	uncultured virus	59	57	4018	Complete RdRp
Serbia reo-like virus 2_Str. 154147	MT822190	*Cimex lectularius*	*Reoviridae*	Parry’s Lagoon virus	98	49	3922	Complete RdRp
Serbia picorna-like virus 2_Str. 153396	MT822188	*Cimex lectularius*	*Picornavirales*	Hubei picorna-like virus 31	73	41	9931	Complete CDS

## Supplementary Materials

The following are available online at https://www.mdpi.com/1999-4915/12/9/975/s1, Table S1 The pool size, collecting sites and date of the arthropods collected from Belgrade, Table S2: The primers used for confirmation of the viruses detected in RNA-seq sequencing, Table S3: Primers used for amplification of the complete CDS or RdRp gene of the novel viruses, Table S4: Reference sequences used in phylogenetic analysis, Table S5: Known viruses identified in this study, Table S6: Positive samples for identified viruses.
